# Effects of a blend of chestnut and quebracho tannins on gut health and performance of broiler chickens

**DOI:** 10.1371/journal.pone.0254679

**Published:** 2022-01-21

**Authors:** Enzo A. Redondo, Leandro M. Redondo, Octavio A. Bruzzone, Juan M. Diaz-Carrasco, Claudio Cabral, Victorino M. Garces, Maximo M. Liñeiro, Mariano E. Fernandez-Miyakawa

**Affiliations:** 1 Instituto de Patobiología Veterinaria, Instituto Nacional de Tecnología Agropecuaria (INTA), Hurlingham, Buenos Aires, Argentina; 2 Consejo Nacional de Investigaciones Científicas y Técnicas (CONICET), Ciudad Autónoma de Buenos Aires, Argentina; 3 EEA Bariloche, Instituto Nacional de Tecnología Agropecuaria, Bariloche, Río Negro, Argentina; 4 Silvateam S.A., Ciudad Autónoma de Buenos Aires, Argentina; 5 Granja Tres Arroyos S.A., Capilla del Señor, Buenos Aires, Argentina; Foshan University, CHINA

## Abstract

Antimicrobial restrictions prompted the search for cost and biologically effective alternatives to replace antimicrobial growth promoters (AGPs) in food-producing animals. In addition, the efficacy of this alternatives needs to be contrasted in field/commercial trials under different challenge conditions. However only a few studies describing the impact of tannins or others AGP-alternatives in commercial poultry production conditions are actually available. The aim of the present work is to study how the inclusion of a blend of chestnut and quebracho tannins can affect broiler productive performance and health under commercial conditions. Three experiments with different approaches were conducted: (1) a trial comparing the effects of both additives (tannins vs AGP) on different commercial farms at the same time; (2) the follow-up of one farm during an entire productive year; and (3) an experimental trial using a *C*. *perfringens* challenge model in broiler chickens. Although productive results from field trials were similar among treatments, evaluations of gut health indicators showed improvements in the tannins treated flocks. Frequency and severity of intestinal gross lesions were reduced in jejunum (42% vs 23%; p<0.05–1.37 vs. 0.73; p<0.01, respectively) and ileum (25% vs. 10%; p<0.0.5–1.05 vs. 0.58; p<0.01) in tannins treated birds. Results from 16S studies, show that cecal microbiota diversity was not differentially affected by AGPs or tannins, but changes in the relative abundance of certain taxa were described, including *Lactobacillus* and *Bifidobacterium* groups. Results from experimental *C*. *perfringens* necrotic enteritis showed that tannins treated birds had reduced incidence of gross lesions in jejunum (43.75 vs. 74.19%; p<0.01) and ileum (18.75% vs. 45.16%; p<0.05) compared with control. These results suggest that AGPs can be replaced by tannins feed additives, and contribute in the implementation of antimicrobial-free programs in broilers without affecting health or performance.

## Introduction

Restrictions on the use of antimicrobials have led to an increase of intestinal health problems in broiler chickens and thus reduced profitability for farmers [1]. A clear example is the increased incidence of *C*. *perfringens* necrotic enteritis (NE) in countries where the use of antimicrobial growth promoters (AGPs) has been banned [2, 3]. This situation not only highlights an excessive dependency of modern animal production on antimicrobials, but also prompted the search for cost and biologically effective alternatives [4, 5]. A large and diverse group of potential alternatives has been investigated and developed over the last years to replace AGPs. Considering that the ideal AGP alternative should improve productive efficiency and promote animal health, plant phytochemicals represent a group of promising candidates [5]. Phytochemicals are natural compounds derived from plant tissues, which include a wide variety of molecules such as tannins, terpenoids, alkaloids and flavonoids, many of which have been found to have an extensive arrangement of biological activities including antimicrobial, antioxidant and anti-inflammatory properties [4–6]. Several works described the potential beneficial effect of phytochemicals, which include maintenance of gut integrity, promotion of beneficial bacteria growth and reduction of negative consequences of bacterial infections [5–7].

Among phytochemicals, tannins stand out for their role as alternative to AGPs in poultry production [7]. In broiler chickens, several tannins have proven to improve growth performance [8] and reduce the detrimental effects of *C*. *perfringens* infection [9]. Previous works from our group, describe that hydrolysable tannins derived from extracts of chestnut tree (*Castanea sativa*) and condensed tannins obtained from quebracho trees (*Schinopsis lorenzii*) have antimicrobial and antitoxin activities against *C*. *perfringens* [10, 11]. In fact, it has been shown that they improve gut health and productive parameters under experimental [12, 13] and commercial conditions [14]. In addition, available data suggest that their use does not contribute to selection and spread of antimicrobial resistance, since pathogenic bacteria such as *C*. *perfringens* do not generate resistance against these tannins even after long term exposure [11]. Besides experimental trials describing the effects of chestnut and quebracho tannins, efficacy should be contrasted in field/commercial trials under different challenge conditions. However, to our knowledge, only a few studies describing the impact of tannins or others AGP-alternatives in commercial poultry production conditions are available in the scientific literature, and these works reported variable results [15–18]. In order to overcome limitations in the study of field application of potential AGP alternatives including chestnut and quebracho tannins, multiple approaches are needed to identify products with high effectiveness rate to reduce antimicrobial needs in poultry industry. Therefore the objective of the present report was to study how the inclusion of a blend of chestnut and quebracho tannins can affect broiler productive performance, intestinal health and cecal microbiota in comparison with conventional AGPs.

## Materials and methods

### Study design and locations

**Fig 1:**
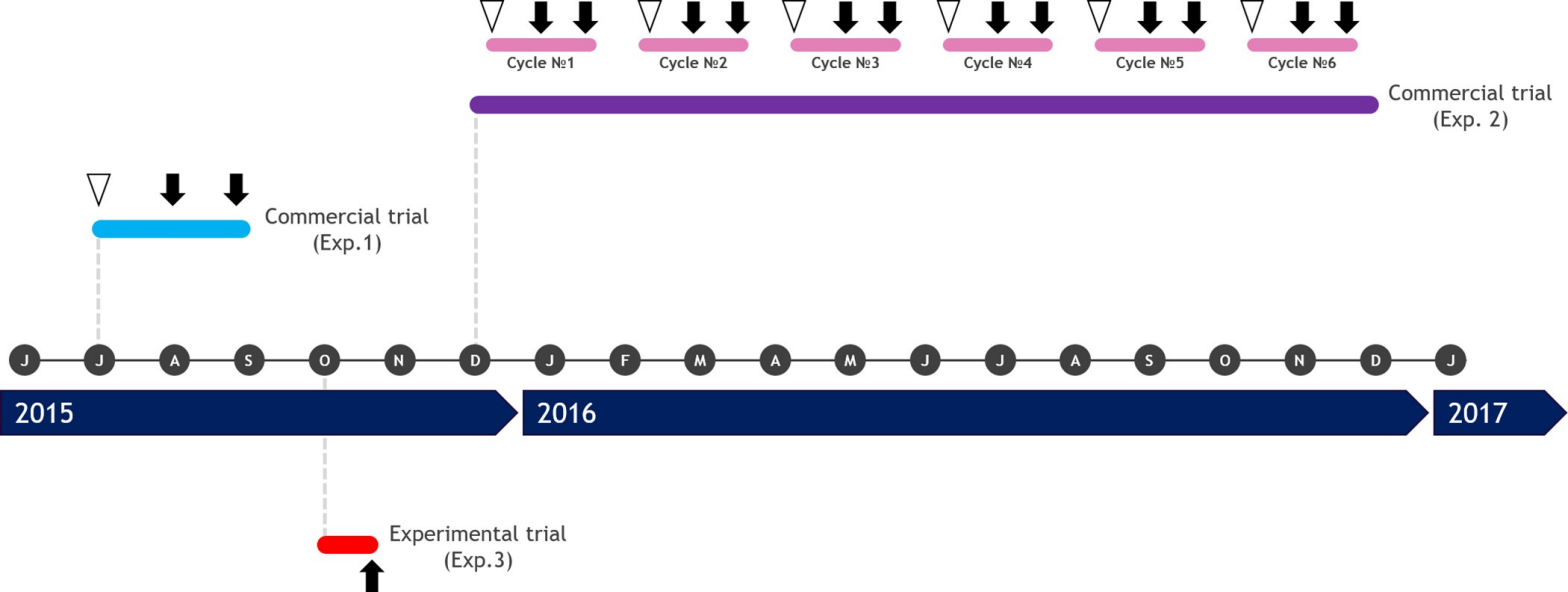
Experimental design and time course of the included experiments. Experiment numbers represent the order in which they were designed and planned. White triangles indicate farms visits to control overall conditions of the birds at arrival. Black arrows indicate necropsies for gross lesions inspection and sample collection.

In order to study outcomes of the addition of chestnut and quebracho tannins compared with AGP, three experiments with different approaches were conducted: (1) a trial comparing the effects of both additives (tannins vs AGP) on different commercial farms at the same time; (2) the follow-up of one commercial farm during an entire productive year; and (3) an experimental trial using a *C*. *perfringens* challenge model in broiler chickens. Field trials were conducted in commercial broiler chicken farms located in the province of Buenos Aires, Argentina. Farms were selected within a radius of 200 km from the Instituto de Patobiologia Veterinaria, CICVyA–INTA among voluntary producers. To be included in the study, each farm was required to have 2–6 houses, with similar stocking densities, surface areas, feeding systems, water equipment and ventilation systems. The capacity of the houses ranged from 10,000 to 20,000 broiler chickens per flock. Selected farms were working under contract with the same integrated chicken company, sharing same hatchery, feed mill and slaughterhouse as well as management practices. Experimental NE challenges were performed in biosafety level 2 facilities located in the Veterinary and Agriculture Research Center (CICVyA-INTA), with controlled temperature and humidity and automated ventilation system. Fig 1 shows time relations among described experiments.

### Institutional Animal Care and Use Committee (IACUC) approval

Experimental and field studies presented here were performed in accordance with the ARRIVE guidelines (https://www.nc3rs.org.uk/arrive-guidelines). The experiments were approved by the Institutional Animal Care and Use Committee of the Veterinary and Agriculture Research Center -INTA, under protocol number 20/2010.

### Plant material

In the present study, chestnut and quebracho extracts were included as a tannin blend commercialized by Silvateam Spa. as powder (SILVAFEED/NUTRI-P). Chestnut extract (*Castanea sativa*) contains 72% tannins principally characterized by the presence of hydrolysable ellagitannins, and was obtained by hot water extraction from the wood of trees grown in the region of San Michele di Mondoví (Italia). Red quebracho extract (*Schinopsis lorenzii*), contains 80% tannins mainly represented by profisetinidin condensed tannin, was also obtained by hot water extraction from trees originated in the region of Gran Chaco (Argentina). A detailed description of the composition of both tannin extracts was previously reported by Molino et al. [19].

### Experiment 1

A prospective study was conducted in 6 commercial broiler chicken farms that met the conditions described before. During this study, chickens were fed in four phases: a pre-starter diet (days 1–14), starter diet (days 15–21), finisher diet (day 22–35) and withdrawal diet (day 36 –clear), nutritional information is summarized in Table 1. Each farm was randomly assigned to one of two treatments: (1) conventional AGP program or (2) tannins-based AGP free program. Birds raised under conventional protocol of the company were fed with commercial diets supplemented with Enramycin (10 g/Tn) as AGP, while in the tannins protocol broiler feed was supplemented with a blend of chestnut and quebracho tannins (SILVAFEED/NUTRI-P 1 Kg/Ton). Both groups were raised similarly, following the conventional management practices as per their feed mill and hatchery guidelines, basically Cobb management handbooks (CoBB-Vantress, 2013). No specific recommendations pertaining to water acidification and brooding were given, and therapeutic antimicrobials or anticoccidial drugs were prescribed by the company veterinarian when needed. Each farm was visited at 1, 21 and 42 days bird’s age. Visits on day 1 were organized to control chicken’s general health conditions at the beginning of every producing period. Parameters such as quality of anticoccidial vaccination, based on the percentage of chicks with the presence of pink dye on the head, crop filling and cloacal temperature were monitored. Results from the 24 h-visits will not be presented in this paper.

**Table 1:** Dietary composition and nutrient levels.

**Ingredients (%)**	**Pre-starter**	**Starter**	**Grower**	**Withdrawal feed**
Corn	63.124	58.573	58.752	61.661
Soybean meal	25.658	25.784	23.521	21.550
Corn gluten meal	3.774	-	-	-
Meat meal	3.500	4.873	3.500	3.500
Soy oil	1.000	1.775	2.100	2.200
Mono/bicalcium phosphate	0.624	-	0.265	0.115
Lysine 50%	0.582	0.303	0.205	0.195
Salt	0.402	0.231	0.255	0.254
Liquid methionine	0.200	-	-	0.200
Shell poder	0.182	-	0.214	0.174
Powder methionine	0.149	0.313	0.229	0.043
Choline 75%	0.100	0.090	0.080	0.070
Mineral premix	0.100	0.100	0.080	0.040
Vitamin premix	0.100	0.100	0.080	0.040
Mycotoxin sequestrant	0.100	-	-	-
Sodium bicarbonate	0.100	0.100	0.100	0.100
Threonine	0.095	0.078	0.039	0.005
Anticoccidial	0.060	0.060	0.060	0.060
Protease	0.020	-	-	-
Phytase	0.020	0.020	0.020	0.020
Antioxidant	0.010	-	-	0.010
Extruded soybean	-	4.500	5.500	5.500
Wheat	-	3.000	4.900	4.263
**Calculated nutrients**	**Pre-starter** ^**1**^	**Starter** ^**1**^	**Grower** ^**2**^	**Withdrawal feed** ^**3**^
Metabolizable energy (Kcal/kg)	3.015	3.110	3.160	3.200
Crude protein (%)	21.100	20.370	19.250	19.080
Dig. Lysine (%)	1.200	1.100	0.990	0.970
Dig. Methionine (%)	0.600	0.578	0.481	0.451
Dig. Met + Cys (%)	0.900	0.836	0.733	0.717
Total phosphorus (%)	0.750	0.720	0.684	0.646
Available phosphorus (%)	0.480	0.440	0.420	0.390
Calcium (%)	0.850	0.840	0.800	0.750

1 Vitamins and minerals provided per kg of diet: 12,000 IU vitamin A; 4,500 IU vitamin D3; 60 IU vitamin E; 4 mg vitamin K; 3 mg vitamin B1; 15 mg vitamin B2; 6 mg vitamin B6; 30 μg vitamin B12; 200 μg biotin; 2.5 mg folic acid; 70 mg niacin; 25 mg pantothenic acid. 120 mg Mn; 100 mg Zn; 45 mg Fe; 20 mg Cu; 1 mg I; 0.3 mg Se.

2 Vitamins and minerals provided per kg of diet: 9,600 IU vitamin A; 3,600 IU vitamin D3; 48 IU vitamin E; 3.2 mg vitamin K; 2.4 mg vitamin B1; 12 mg vitamin B2; 4.8 mg vitamin B6; 24 μg vitamin B12; 160 μg biotin; 2 mg folic acid; 56 mg niacin; 20 mg pantothenic acid. 96 mg Mn; 80 mg Zn; 36 mg Fe; 16 mg Cu; 0.8 mg I; 0.24 mg Se.

3 Vitamins and minerals provided per kg of diet: 4,800 IU vitamin A; 1,800 IU vitamin D3; 24 IU vitamin E; 1.6 mg vitamin K; 1.2 mg vitamin B1; 6 mg vitamin B2; 2.4 mg vitamin B6; 12 μg vitamin B12; 80 μg biotin; 1 mg folic acid; 28 mg niacin; 10 mg pantothenic acid. 48 mg Mn; 40 mg Zn; 18 mg Fe; 8 mg Cu; 0.4 mg I; 0.12 mg Se.

### Experiment 2

Based on productive performance, structural and sanitary conditions, one of the farms included in “Experiment 1” was selected to compare the global outcome of using AGPs or tannins based-programs on broiler performance and health over a one-year period. In the selected farm, each of six tunnel ventilated broiler houses (~20,000 birds) was randomly assigned to one of two treatments: (1) conventional AGP program (AGP rotation: Bacitracin 50 g/Tn, Avilamycin 10 g/Tn, Enramycin 10 g/Tn), or, (2) tannins-based AGP free-program (SILVAFEED/NUTRI-P 1 Kg/Ton). This assignment was kept during 6 production cycles over a 5- or 7-week period according to commercial requirements. A total of 720,000 mixed sex birds were included in this trial. General management practices other than antimicrobial use were like those described for “Experiment 1”. Similarly, during each productive cycle this farm was visited at 1, 21 and 35/42 days. All barns were monitored, and general health of the birds was controlled as described for experiment 1.

### Data and sample collection from field trials

#### Productive performance

In both experiments, zootechnical performances of the flocks included in this study were provided by the poultry producer. On each house included in field trials, 50 birds were randomly selected to determine weekly body weight (BW), additional body weight records were obtained from slaughterhouse. Mortality was obtained weekly and feed consumption was obtained at the conclusion of each cycle. Feed conversion (FCR) was calculated as the feed to gain ratio. The gain, feed intake, and feed conversion were corrected for dead birds. European Poultry Efficiency Factor (EPEF) values were calculated for overall growth period using the following standard formula: EPEF = [(BW in Kg x % Livability)/ (Age in days x FCR)] x 100.

#### Necropsy and sample collection

A similar methodology was used for both experiments. On day 21, 10 healthy male birds per barn were randomly selected. Birds were euthanized by cervical dislocation and complete necropsy was immediately performed for examination of gross lesions. During necropsies, the small intestine segments and ceca were carefully observed, and gross lesions were recorded and scored blindly by two experienced pathologists as described by Cooper and Songer [20]. Footpad lesions were determined by visual inspection and palpation and classified using a 5-point score system based on the presence, size and severity of lesions [21]. Segments of 2-cm in length from the mid-points of the duodenum, jejunum, and ileum were cut, flushed with cold saline, and immediately preserved in 10% phosphate-buffered formalin for histomorphological analysis [22]. Additionally, during experiment 2, cecal contents were transported at 4°C and stored at -80°C for DNA extraction and determination of cecal microbiota. During rearing periods, all groups were routinely supervised, and clinical examinations were performed regularly. Additional visits were conducted if an increased mortality rate was observed by the producer. In those circumstances, necropsies were performed on moribund and dead birds. Finally, birds were visited on the day before slaughter (day 35 or 42). Necropsies, lesion scoring, and intestinal sampling were repeated exactly as described for day 21.

### Experiment 3

In order to evaluate the response to an outbreak of NE in chickens under an AGP-free program based on tannins additives, further trials were performed under experimental conditions using an *in vivo* infection model for necrotic enteritis (NE) by *C*. *perfringens* in chickens for fattening [20].

#### Birds and housing

Experimental NE challenges were performed with 175 male Cobb broiler chickens. Birds were obtained as 1-day-old chicks from the same commercial hatchery which provides birds for experiments 1 and 2. On arrival day, birds were randomly divided in 15 groups of 11–12 chicks each. All treatment groups were housed in the same room, placed in pens (1.5 × 1.5 × 0.8 m) made of 0.55 mm wire mesh and hardboard pieces covering the lower part of the mesh to separate them. Commercial wood shavings were used as bedding material and maintained until the end of the trial. Commercial starter feed described before (Table 1) was placed in galvanized steel trays and given ad-libitum, the same for water. For experimental treatments, the commercial feed was mixed with different tannin-based additives (final concentration 1 Kg/Ton), and each group of birds was randomly assigned to one of 5 experimental treatments: (1) negative control: feed without tannin additives, NE unchallenged; (2) positive control: feed without tannins additives, NE challenged; (3) chestnut: feed with chestnut based additives, NE challenged; (4) quebracho: feed with quebracho based additives, NE challenged; (5) mix: commercial mix of chestnut and quebracho (SILVAFEED–NUTRI P) additive, NE challenged).

#### *In vivo* NE model

A *C*. *perfringens* strain (*cpa+*, *cpb2-*, *netB+*, *TpeL-*) isolated from a natural outbreak of broiler NE was used as inoculum to challenge broilers. The strain was prepared by streaking glycerol aliquots onto blood agar plates and grow under anaerobic conditions (5% H_2_:5% CO_2_:90% N_2_) for 18 h at 37°C. Then, 1–2 colonies were transferred into 10 ml cooked meat medium (CMM) and further incubated for 18 h at 37°C. This culture was inoculated in 100 ml fluid thioglycollate broth (FTG) and cultured as before. After 18 hours, the FTG culture was diluted 1:10 in CMM and incubated as before. One hundred ml of the last CMM culture was used as inoculum for 1 L of FTG medium, after 18 h of incubation this culture reached a concentration of 7–9 × 10^8^ cfu/ml. This last FTG culture was used as inoculum and procedure was repeated for each dose used during the challenge (total challenge doses, n = 6). On day 15, birds were fasted for 12 h prior to the initial challenge on day 16. Challenge was performed orally by mixing *C*. *perfringens* FTG culture with commercial feed (1:1 v/w) and given to birds twice a day on days 16, 17 and 18. Uneaten feed was replaced before each subsequent challenge. All groups were routinely supervised and clinical examinations were performed at least twice a day during these experiments. Since death was not an endpoint of this experiment, when chickens displayed respiratory distress, injuries, were reluctant to move, or showed severe weight loss, they were humanely euthanized according to the approved protocol. On day 19, some birds from each group (n = 5) were euthanized by cervical dislocation and necropsy was performed immediately for examination of intestinal gross lesions as described for the field trials. To confirm the identity of intestinal lesions, tissue samples were taken from each bird with gross lesions. Samples were kept refrigerated or in buffered formalin solution for both bacteriological and histopathological diagnosis.

### Microscopic analysis of digestive tracts

Intestinal segments were fixed for at least 48 h in 10% phosphate-buffered formalin for histological analysis. The processing consisted of serial dehydration, clearing, and impregnation with wax. Tissue sections, 5 μm thick (3 cross-sections from each sample), were cut by a microtome and were fixed on slides. A routine staining procedure was carried out using hematoxylin and eosin. Histological analysis of the intestinal samples was done using standard light microscopy (Microscope NIKON ECLIPSE 80i Co., Ltd, Japan), a camera (DS-Fi1c) and a computer-based image analysis system (ImageJ—version 1.53c. National Institutes of Health). Villi fusion, presence of coccidia, necrotic debris, capillary congestion, etc. were considered. For histomorphometric studies, well-oriented crypt-villus units were selected in triplicate for each intestinal cross-section for each sample. The criterion for villus selection was based on the presence of intact lamina propria. Villus height (VH, from the tip of the villus to the top of the lamina propria), crypt depth (CD, from the base to the region of transition between the crypt and villus) and the thickness of the muscularis mucosae in the duodenum, jejunum and ileum were determined. Measurements of 10 complete villi for VH and associated crypts for CD were taken from each segment and the average of these values was used for statistical analysis [21].

### Cecal microbiota

#### DNA extraction

Metagenomic DNA was isolated from 300 mg of pooled cecal contents using QIAamp DNA Stool Mini Kit (Qiagen, Hilden, Germany) following manufacturer’s instructions. DNA concentration and quality were assessed in NanoDrop ND-1000 spectrophotometer (NanoDrop Technologies, DE, USA). DNA was stored at −20°C until further analysis.

#### 16S rRNA gene library preparation and high-throughput sequencing

One-step PCR was performed according to the 16S rRNA metagenomics protocol for MiSeq platform (Illumina, San Diego, CA, USA). The 16S rRNA gene V3V4 regions were amplified using universal primers (forward 341F 5′ CCT ACG GGN GGC WGC AG 3′, reverse 806R 5′ GAC TAC HVG GGT ATC TAA TCC 3′) with standard adapter sequences attached for barcoding and multiplexing. The resulting amplicons (~560-bp) were confirmed by gel electrophoresis on a 1.5% agarose gel stained with Gelred. The amplicons were purified using Agencourt AMPure XP System (Beckman Coulter, Beverly, MA, USA) and their concentrations estimated by the Quant-iT PicoGreen dsDNA assay (Thermo Fisher Scientific, Waltham, MA, USA), and Index PCR of the purified products was performed using Fast Start^™^ High Fidelity PCR System (ROCHE) following a standardized protocol. In order to reduce unbalanced and biased base compositions, 10% of PhiX control library was spiked into the amplicon pool. 16S rRNA V3V4 libraries were sequenced on Illumina MiSeq using a paired-end 250-bp protocol and v2 reagents at Genomic Unit of Biotechnology Institute, INTA (Hurlingham, Buenos Aires, Argentina). All sequence data were deposited in the Center for Open Science database under the accession DOI 10.17605/OSF.IO/YNAWT (https://osf.io/ynawt/).

#### Bioinformatic and statistical analysis of microbial community profiles

The sequences were processed using QIIME2 software version 2021.4 [23]. The primer sequences were trimmed from the raw reads and sequences were subsequently denoised using the DADA2 plugin in QIIME2. In order to obtain denoised amplicon sequence variants (ASVs), 3’ ends of the reads were subjected to further trimming based on the quality score distribution, as well as quality filtering and chimera removal. The taxonomy was assigned using a naive Bayesian classifier trained on the Green Genes 99% full-length 16S rRNA gene sequence database. Alpha diversity parameters of the microbial communities were calculated in QIIME2 through richness (number of ASVs), entropy (Shannon’s index), phylogenetic diversity (Faith’s index) and evenness (Simpson’s evenness index) using a rarified table as input. Distributions were compared trough non-parametric Mann-Whitney test. The bacterial composition of cecal microbiota was analyzed using Statistical Analysis of Metagenomic Profiles (STAMP) software version 2.1.3 [24]. Relative abundances between groups were compared by non-parametric two-tailed White’s test at species level of classification. STAMP was set to dismiss taxa detected in a single sample. Correlations between productive efficiency indicators and taxonomic relative abundance at the phylum and genus levels were determined using Spearman correlation coefficients, only r index with p-values<0.05 were considered.

### Estimation of parameters to describe growth

To study the growth, a Gompertz growth curve [25] was fitted to the data and the parameters of the curve (initial weight, growth rate, and final weight) were compared between treatments. Over that base model, we added three more parameters, described in S1 Table in S1 File (autocorrelation coefficient, error intercept, and error slope) to compensate for measurement errors, and autocorrelation, as described below.

An autoregressive model was used to compensate for the autocorrelation caused by the repeated measures on the same individual, so the growth curve was (Eq 1):

$$dW/dt=r\;W\;log\left(W/W_{f}\right)$$ (1)

with initial conditions *W*_*(t = 0)*_ *= W*_*o*,_ being W the weight in grams of the animal, and *r* its growth rate in 1/days, W_o_ is the weight at the beginning of the experiment. Given that the animals were measured repeated times, *W*_*i*_ represents the estimated weight at the measurement time *i*, which was corrected using an autoregressive model as follows (Eq 2):

$$\begin{array}{l} W_{i}=W_{t=i}+\varphi \;\left(W_{ot=i‐1}‐W_{t=i‐1}\right)+\varepsilon \\ with\;\varepsilon ∼N(W_{i},\;\sigma_{o}+\sigma_{s}W_{i}) \end{array}$$ (2)

with t being time, i-1 being the time of previous measurement, W_o_ the observed weight, and φ an autoregressive coefficient, ɛ are the errors which are normally distributed with mean zero, as we observed that errors increased with the weight of the animal, then we used a standard deviation which increases linearly with the expected weight, being *σ*_*o*_ the intercept and *σ*_*s*_ its slope.

All the parameters and information indexes of the proposed models were calculated using Markov Chain Monte Carlo (MCMC) methods with the Metropolis-Hastings algorithms. The Monte Carlo calculations were performed with the python programming language and the pymc version 2.4 for Monte Carlo methods [26].

### Statistical analysis

For field trials the statistical unit was the flock in all analyses, and AGPs treated groups were considered as control for analysis. Performance data were corrected with mortality and subjected to one-way ANOVA analysis. Necrotic enteritis lesion scores were statistically analysed using a two-tailed Fisher exact test to assess the difference between proportions of pathological lesion development between treatment groups in both field trials and the experimental challenge trial. Paired t tests were used for analysis of lesion scores and histomorphometric determinations. Described analysis were performed with GraphPad Prism software version 5.01. Differences were considered significant at P ≤ 0.05.

## Results

### Experiment 1

#### Productive performance

No significant differences were observed in live weight (2.621 kg vs. 2.523 kg), feed conversion (1.71 vs. 1.73), mortality (3.9% vs. 3.4%) or EPEF (300 vs. 288) between farms under AGP and tannins-based programs respectively.

#### Necropsy findings and intestinal structure

Frequency and severity of intestinal gross lesions were reduced in birds from tannins treated farms (Table 2). Jejunal gross lesions were present in 23.3% of the necropsied birds compared with the 42.5% in the AGP farms (p<0.05), similar results were observed in the ileum (10% vs 25%, p<0.05), although differences were not significant, duodenal lesions were also reduced (24.4% vs. 30%). Those differences were more evident during the starter period (day 21). Similar results were obtained regarding lesion severity, gross lesion score were reduced in jejunum (0.73 vs 1.37, p<0.01) and ileum (0.58 vs. 1.05, p<0.005), duodenal scores were also reduced although differences were not significant (0.87 vs 1.15). No differences were observed in the frequency of footpad lesions and presence of undigested feed in distal portions of digestive tract. Histomorphological measurements for duodenal, jejunal and ileal sections were similar in both treatments; results from these determinations are presented in Table 3.

**Table 2:** Frequency and score of intestinal gross lesions^1^ (Experiment 1).

	AGPs		Tannins	
	Frequency (%)	Score (mean)	Frequency (%)	Score (mean)
**Day 21**				
Duodenum	30	1.30	28	1.08
Jejunum	35	1.35	18 *	0.56 *
Ileum	35	1.20	12 *	0.58 *
**Day 42**				
Duodenum	30	1.00	20 *	0.62
Jejunum	50	1.40	30	0.95
Ileum	15	0.90	7.50	0.60 *
**Total**				
Duodenum	30	1.15	24.4	0.87
Jejunum	42.5	1.37	23.3 *	0.73 **
Ileum	25	1.05	10 *	0.58 **

*: p<0.05

**: p<0.01.

^1^ Intestinal gross lesion score: 0: no apparent gross lesions; 1: removable fibrin deposit; 2: isolated focal necrosis or ulceration (1 to 5 foci); 3: multiple focal necrosis or ulceration (6 or more foci); 4: extensive areas of necrosis; 5: diffuse necrosis, presence of attached pseudomembrane).

**Table 3:** Intestinal histomorphometry (Experiment 1).

		AGPs	Tannins	p-values
**Day 21**				
Duodenum	Villus height (μm)	1874 ± 160	1709 ± 76	0.336
	Crypt depth (μm)	263 ± 64	241 ± 51	0.695
	Villus/Crypt ratio	7.71 ± 1.06	7.81 ± 0.9	0.950
Jejunum	Villus height (μm)	1061 ± 103	812 ± 80	0.143
	Crypt depth (μm)	167 ± 12	196 ± 33	0.438
	Villus/Crypt ratio	6.56 ± 1.0	4.71 ± 0.96	0.303
Ileum	Villus height (μm)	539 ± 41	503 ± 27	0.503
	Crypt depth (μm)	126 ± 19	134 ± 3	0.595
	Villus/Crypt ratio	4.43 ± 0.37	3.84 ± 0.2	0.197
**Day 35–42**				
Duodenum	Villus height (μm)	1958 ± 38	1998 ± 53	0.545
	Crypt depth (μm)	241 ± 11	204 ± 8	0.009
	Villus/Crypt ratio	8.53 ± 0.34	10.6 ± 0.67	0.011
Jejunum	Villus height (μm)	977 ± 60	1168 ± 95	0.098
	Crypt depth (μm)	127 ± 6	149 ± 10	0.058
	Villus/Crypt ratio	7.9 ± 0.5	8.79 ± 0.97	0.423
Ileum	Villus height (μm)	512 ± 18	525 ± 26	0.669
	Crypt depth (μm)	106 ± 6	148 ± 14	0.012
	Villus/Crypt ratio	4.88 ± 0.24	3.7 ± 0.2	0.04

### Experiment 2

#### Productive performance

**Fig 2:**
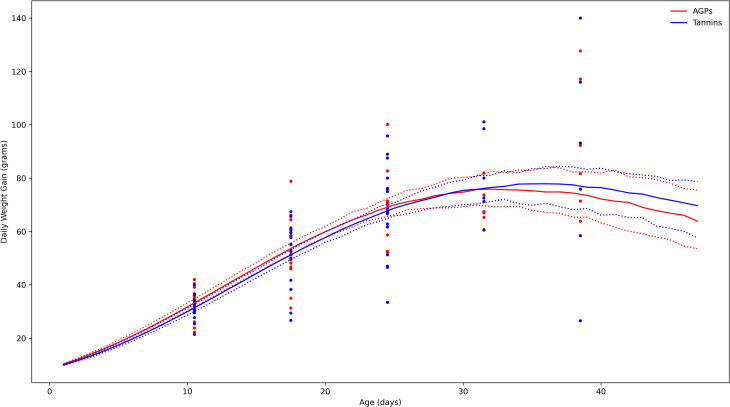
Daily weight gain derived from Gompertz equation. Solid lines represent estimated mean daily weight gains at different ages (R*Wf) for tannins (blue) and AGPs (red) treated flocks, dotted lines represents CI at 95% for the growth curve after 100000 montecarlo simulations. Circles represent average daily gains estimated from observed weekly weights (see Table 4).

Productive outcomes were similar among flocks included in both treatments. No statistically significant differences were observed between AGP and tannin treatments in weekly (Table 4) or final bodyweight either at 5 (1.452 Kg vs. 1.491 Kg, respectively) or 7 weeks (2.538 Kg vs. 2.608 Kg), flock uniformity expressed as CV (12,9% vs. 12,5%), feed conversion (1.767 vs. 1.762) or EPEF values (268 vs. 271). During this experiment and depending on commercial requirements, birds were slaughtered at different ages. Therefore, potential growth and weight gains were estimated by Gompertz equation. According to this model, tannins treated birds will have higher growth potential and growth rate may decrease at a slower rate compared with the AGPs treated groups (Fig 2) (S1 Table in S1 File).

**Table 4:** Growth performance in broilers under AGPs or tannins programs (experiment 2).

	AGPs	Tannins	p-values
Age	Body weight (g.)^1^		
7 days	159.18 (14.96)	153.41 (15.03)	0.948
14 days	402.35 (37.82)	383.57 (37.58)	0.959
21 days	785.34 (73.82)	750.79 (73.57)	0.947
28 days	1271.88 (119.55)	1229.68 (120.50)	0.858
35 days	1801.96 (169.38)	1766.09 (173.07)	0.680
42 days	2315.62 (217.66)	2305.73 (225.96)	0.514

^1^ Values are expressed as mean (SD).

**Fig 3:**
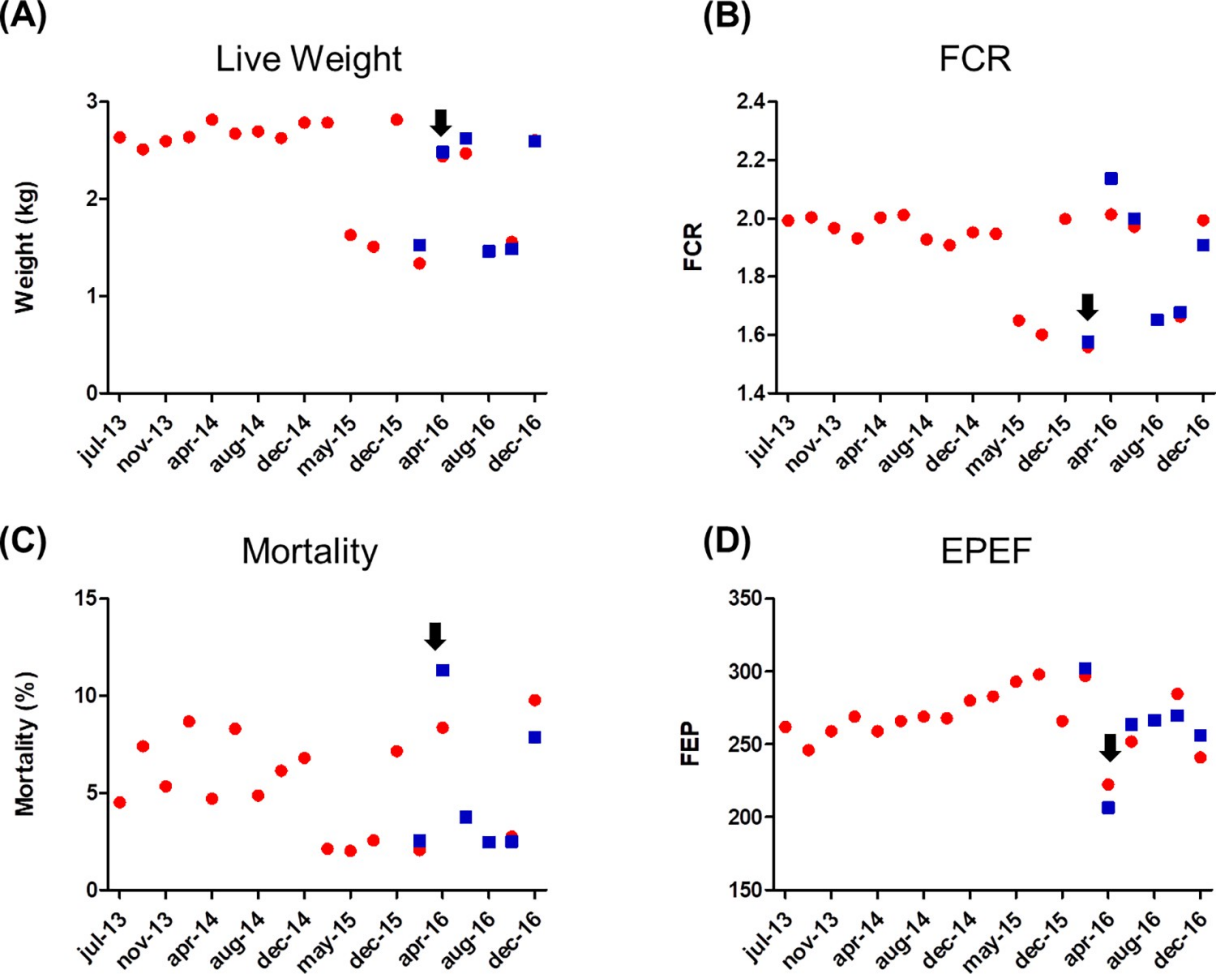
Productive historical records and results from experiment 2. A) Live body weight at the end of the productive cycle; B) Feed conversion ratio (FCR); C) Mortality; and D) European Poultry Efficiency Factor (EPEF). The period included in the present study starts from December 2015 (dec-15) to December 2016 (dec-16); red dots represent data from flocks under AGPs program and include data from previous productive cycles; blue squares represent data from AGPs free/tannins based program. Black arrow indicates the occurrence of a respiratory disease outbreak.

Tannins/AGP free treated flocks showed a mild reduction (no significant) in total mortality (4.169% vs. 3.823%) and the mortality to the first week (0.873% vs. 0.721%). During the second productive cycle a respiratory disease outbreak occurred in a barn under tannin treatment, increasing global tannins mortality (11.3%) compared to AGP (8.3%) treated flocks. Since these flocks were treated with antimicrobials, data from this period were omitted from analysis. Productive historical records and results from the studied period are summarized in Fig 3.

#### Necropsy findings

A total of 180 birds (with an average of 10 birds per flock/time) were evaluated. A description of intestinal lesions from this experiment is shown in Table 5. In general, a mild reduction of gross intestinal lesions was observed among the tannin treated flocks examined on starter (day 21) and finisher periods (days 35 or 42). In contrast, lesion scores were higher in jejunum of birds from the tannin treated flocks during finisher periods. The condition of footpads deteriorated towards slaughter age, and despite this tendency was similar in both treatments, the tannin treated flocks, showed reduced frequency (77.8% vs 64.4%; p<0.05) of footpad lesions and reduced severity as represented by the mean score lesion (1.067 vs 0.766; p<0.0001).

**Table 5:** Frequency and score of intestinal gross lesions^5^ (Experiment 2).

	AGPs		Tannins	
	Frequency (%)	Score (mean)	Frequency (%)	Score (mean)
**Day 21**				
Duodenum	2	0.28	2	0.24
Jejunum	16	0.74	4 *	0.44 **
Ileum	4	0.62	6	0.62
**Day 35–42**				
Duodenum	12.5	0.6	7.5	0.32 ***
Jejunum	22.5	0.85	22.5	1.12 *
Ileum	12.5	0.65	ND	ND
**Total**				
Duodenum	6.7	0.42	4.5	0.27
Jejunum	18.9	0.78	12.2	0.74
Ileum	7.8	0.63	3.4	0.64

*: p<0.05

**: p<0.01

***: p<0.001.

ND: no lesions detected.

^1^ Intestinal gross lesion score: 0: no apparent gross lesions; 1: removable fibrin deposit; 2: isolated focal necrosis or ulceration (1 to 5 foci); 3: multiple focal necrosis or ulceration (6 or more foci); 4: extensive areas of necrosis; 5: diffuse necrosis, presence of attached pseudomembrane).

#### Histomorphology

At day 21, reduced villus height in duodenum (p< 0.05) and jejunum (p< 0.001) were observed in tannin treated flocks, while ileal villi were higher (p< 0.001). In contrast, on finisher period villus height was increased in duodenum (p< 0.001) and jejunum (p<0.001). The means of duodenal, jejunal and ileal villus height, crypt depth, and villus:crypt ratio are presented in Table 6.

**Table 6:** Intestinal histomorphometry (Experiment 2).

		AGPs	Tannins	p-values
**Day 21**				
Duodenum	Villus height (μm)	1602 ± 35	1488 ± 34	0.027
	Crypt depth (μm)	187 ± 7	184 ± 9	0.812
	Villus/Crypt ratio	9 ± 0.3	8.9 ± 0.5	0.906
Jejunum	Villus height (μm)	852 ± 16	765 ± 18	0.0007
	Crypt depth (μm)	152 ± 5	149 ± 6	0.709
	Villus/Crypt ratio	5.87 ± 0.2	5.64 ± 0.3	0.565
Ileum	Villus height (μm)	401 ±15	501 ± 17	<0.0001
	Crypt depth (μm)	121 ± 3	138 ± 4	0.0028
	Villus/Crypt ratio	3.35 ± 0.1	3.78 ± 0.2	0.068
**Day 35–42**				
Duodenum	Villus height (μm)	1654 ± 23	1864 ± 31	<0.0001
	Crypt depth (μm)	144 ± 6	135 ± 6	0.312
	Villus/Crypt ratio	12.64 ± 0.5	15.87 ± 0.9	0.016
Jejunum	Villus height (μm)	946 ± 22	1102 ± 28	<0.0001
	Crypt depth (μm)	158 ± 8	171 ± 5	0.211
	Villus/Crypt ratio	7.27 ± 0.3	6.81 ± 0.2	0.295
Ileum	Villus height (μm)	516 ± 14	480 ± 12	0.065
	Crypt depth (μm)	119 ± 4	118 ± 3	0.790
	Villus/Crypt ratio	4.5 ± 0.1	4.2 ± 0.1	0.165

#### Cecal microbiota

Alpha diversity parameters did not show significant differences between the two treatments (S1 Fig). Except for the Simpson’s index, diversity parameters showed a strong negative correlation with productive efficiency and these correlations were similar among treatments (S2 Table in S1 File). At the phylum level, the microbiota profiles also showed great similarity, with a marked predominance of species belonging to phylum Firmicutes. Relative abundance of the phyla Bacteroidetes and Tenericutes showed a negative correlation with productive efficiency (r: -0.52, p<0.05 and r: -0.57, p<0.05 respectively) while a positive correlation was observed with Firmicutes (r: 0.48, p<0.05), these correlations were stronger within the conventional (AGP treated) groups. No significant differences were observed in the Firmicutes/Bacteroidetes ratio, although it was slightly higher in birds treated with tannins (S2 Table in S1 File). Significant differences were found in the relative abundance of ten bacterial taxa between AGP and tannin treated flocks (p<0.05), while four other taxa showed a trend (p<0.10) (Table 7). Bacterial groups overrepresented in birds treated with AGP include the unclassified members of families *Desulfovibrionaceae* and *Synergistaceae*, genera *Slackia*, *Anaerostipes* and *Clostridium*, and species *Bacteroides coprophilus*, *Megamonas hypermegale* and *Escherichia coli*. On the other hand, in the birds treated with tannins, members of the family *Bifidobacteriaceae*, genera *Butyricimonas*, *Coprococcus*, *Ruminococcus* and species *Lactobacillus helveticus* and *Subdoligranulum variabile* were increased. Among these taxonomic groups, the genera *Butyricimonas* shows a positive correlation with BW and FCR, while the genera *Clostridium* was correlated with decreases in productive efficiency (Table 8). Additional description of cecal microbiota composition taxonomy from phylum to species levels is present in S2 Fig.

**Table 7:** Cecal microbiota composition, AGPs vs. tannins (Experiment 2).

			AGPs		Tannins		
Family	Genus	Species	Relative abundance (%)	SD	Relative abundance (%)	SD	p-values
*Bifidobacteriaceae*	Unclassified		0.20770	0.18275	0.77609	0.60826	0.009
*Coriobacteriaceae*	*Slackia*	Unclassified	0.06236	0.15305	ND	ND	0.001
*Bacteroidaceae*	*Bacteroides*	*coprophilus*	0.30551	0.66617	ND	ND	0.023
*[Odoribacteraceae]*	*Butyricimonas*	Unclassified	0.35844	0.35595	0.79575	0.61195	0.089
*Lactobacillaceae*	*Lactobacillus*	*helveticus*	1.19341	1.09405	2.93944	1.84471	0.020
*Clostridiaceae*	*Clostridium*	Unclassified	0.02676	0.04389	ND	ND	0.001
*Lachnospiraceae*	*Anaerostipes*	Unclassified	0.01892	0.03556	ND	ND	0.001
	*Coprococcus*	Unclassified	0.16601	0.25298	0.54717	0.33977	0.013
	*[Ruminococcus]*	Unclassified	0.39645	0.38623	1.00365	1.01141	0.083
*Ruminococcaceae*	*Subdoligranulum*	*variabile*	0.65751	0.57424	1.23472	0.78919	0.079
*Veillonellaceae*	*Megamonas*	*hypermegale*	0.31234	0.47607	0.02222	0.07025	0.100
*Desulfovibrionaceae*	Unclassified		1.03726	0.62765	0.24365	0.29632	0.006
*Enterobacteriaceae*	*Escherichia*	*coli*	0.13751	0.17889	0.01695	0.04001	0.074
*Synergistaceae*	Unclassified		0.10158	0.17299	ND	ND	0.006

ND: not detected.

**Table 8:** Cecal microbiota composition correlated with productive indicators (Experiment 2).

			BW		FCR		EPEF	
Family	Genus	Species	r	p-values	r	p-values	r	p-values
*Bifidobacteriaceae*	Unclassified		0.012	0.961	-0.084	0.724	0.123	0.604
*Coriobacteriaceae*	*Slackia*	Unclassified	0.139	0.581	0.183	0.439	-0.358	0.120
*Bacteroidaceae*	*Bacteroides*	*coprophilus*	0.213	0.396	0.232	0.322	-0.338	0.144
*[Odoribacteraceae]*	*Butyricimonas*	Unclassified	0.583	0.011	0.585	0.006	-0.333	0.150
*Lactobacillaceae*	*Lactobacillus*	*Helveticus*	0.069	0.784	0.285	0.222	-0.260	0.267
*Clostridiaceae*	*Clostridium*	Unclassified	-0.014	0.953	0.216	0.358	-0.554	0.011
*Lachnospiraceae*	*Anaerostipes*	Unclassified	0.19	0.449	0.185	0.433	-0.199	0.397
	*Coprococcus*	Unclassified	0.155	0.538	0.306	0.189	-0.235	0.317
	*[Ruminococcus]*	Unclassified	0.17	0.5	0.158	0.503	0.062	0.793.
*Ruminococcaceae*	*Subdoligranulum*	*variabile*	-0.44	0.063	-0.466	0.038	0.434	0.055
*Veillonellaceae*	*Megamonas*	*hypermegale*	0.257	0.303	0.247	0.296	-0.172	0.466
*Desulfovibrionaceae*	Unclassified	Unclassified	-0.105	0.677	-0.168	0.478	0.157	0.505
*Enterobacteriaceae*	*Escherichia*	*Coli*	-0.201	0.422	-0.244	0.298	0.365	0.112
*Synergistaceae*	Unclassified	Unclassified	0.209	0.404	0.195	0.408	-0.180	0.445

BW: body weight; FCR: Feed conversion rate; EPEF: European productive efficiency factor.

### Experiment 3

#### Necropsy findings

Tannin treated groups showed a clear reduction in the frequency and score of intestinal lesions observed on day 19, after an experimental challenge with a NE *C*. *perfringens* strain. Birds treated with tannins mix presented lower percentages of gross lesions in jejunum (43.75%, p<0.01) and ileum (18.75%; p<0.05), compared with the results obtained from positive control, 74.19% and 45.16% for jejunum and ileum respectively. Considering the incidence of severe lesions (score >3), significant differences were only observed in the ileum of mix treated birds (S3 Table in S1 File). Reduced lesion score was observed in the jejunum (1.19 vs 1.97; p<0.01) and ileum of mix treated birds (0.63; p<0.05). Chestnut treated groups also showed a reduction in ileum gross lesions (0.58; p<0.05), in comparison with the score registered for the positive control group (1.29). Results from this trial are summarized in Table 9. No mortality was noticed among the challenged groups, and no intestinal gross lesions were detected in the negative control group (no challenge-no additives). Microscopic observation of these lesions revealed foci of necrosis, haemorrhage and epithelial desquamation. In most severe cases, accumulation of fibrinous exudates was observed. Mucosal smears of gross lesions in the small intestine of challenged groups showed abundant short Gram-positive bacilli compatible with *C*. *perfringens*. Bacteriological and molecular probes confirmed the identity of the obtained isolates.

**Table 9:** Frequency and score of intestinal gross lesions^1^ (Experiment 3).

Treatment	Jejunum		Ileum	
	Frequency (%)	Score (mean)	Frequency (%)	Score (mean)
Positive control	74.19	1.97	45.16	1.29
Chestnut	66.67	1.71	16.67*	0.58*
Quebracho	76.67	1.67	43.33	1.00
Tannins mix	43.75**	1.19**	18.75*	0.63*

*: p<0.05

**: p<0.01.

^1^ Intestinal gross lesion score: 0: no apparent gross lesions; 1: removable fibrin deposit; 2: isolated focal necrosis or ulceration (1 to 5 foci); 3: multiple focal necrosis or ulceration (6 or more foci); 4: extensive areas of necrosis; 5: diffuse necrosis, presence of attached pseudomembrane).

## Discussion

Phytochemicals are considered as feed additives in poultry because of their beneficial effects in animal performance [8, 27–29]. Tannin-based products are a specific group of phytochemicals that have been used in commercial operations for many years [5, 14]. However, and despite their extensive use, reports about their application under commercial conditions are limited [6, 14]. In the present study, results from two different field trials show that dietary addition of a blend of chestnut and quebracho tannins at 0.1% (w/w) with no AGPs contributes to achieve similar productive levels when compared in parallel to commonly used AGPs (bacitracin, avilamycin and enramycin).

Results from necropsies, histologic and gut microbiota studies provided evidence that this blend of tannins contributes to improve gut health and reduce intestinal alterations associated with *C*. *perfringens* NE under experimental and field conditions. Our results are similar to those reported by Manelli et al. [27] and Liu et al. [28], where the authors described positive effects on growth rate after the addition of chestnut tannins (1–3 g/kg). In contrast, Jamroz et al. [30] did not find any significant effects on body weight gain and feed conversion in broilers supplemented with chestnut tannins (250 or 500 mg/kg), similar results are described by Brenes et al. [31] in broilers fed with grape pomace (condensed tannins at 1,500 mg/kg) at different levels (15 mg, 30 mg, and 60 mg/kg). Unfortunately, these works only describe trials under experimental conditions which may limit direct comparisons. On the other hand only few works describe evaluations under commercial conditions. A clear exception is the study performed by Gaucher et al. [17]. This work uses a similar experimental design and phytochemicals were also used to control health issues in AGPs free flocks, but in this study the authors described significantly decreased zootechnical performances compared with conventionally raised flocks, which was attributed to a higher impact of infectious diseases outbreaks [17].

Along with the negative productive consequences, an increased incidence of infectious diseases like *C*. *perfringens* NE and *Campylobacter* shedding were reported in association with antimicrobial restrictions [3, 17, 32], resulting in poor zootechnical performance and increased condemnations at slaughter [33, 34]. In our study, clinical necrotic enteritis cases were not identified during farm visits nor informed by farmers, but sub-clinical necrotic enteritis was regularly observed during systematic necropsies of apparently clinically healthy chickens within each flock of both AGP and tannin treated animals. The estimated prevalence of subclinical NE was reduced in the tannin treated groups (10–25%) compared with a 25–45% in AGP groups, concomitantly with an important reduction in the score of intestinal lesions, especially on observations performed on day 21 of age. In contrast to our study, Gaucher et al., reported no clinical or subclinical NE in conventional AGP programs, but the authors observed a prevalence of 30–50% in AGP-free programs based on essential oils [17]. The major difference between the 2 studies was that while essential oils were only included during NE outbreaks in order to reduce negative consequences, in our study tannins additives were applied during the whole productive cycle. Authors described that this treatment contributes to reduce NE associated mortality but failed to handle productive losses. Several reports describe the *in vitro* inhibitory effects of all of these phytochemicals against *C*. *perfringens* [35], but the contrasting results obtained *in vivo* confirm that despite many essential oils or tannins can share certain biological activities such as critical antimicrobial characteristics [5, 10, 13], other biological activities and their combinations are crucial to achieve consistent and reproducible productive results comparable with those obtained with AGPs. Moreover, it probably becomes clearest during an outbreak of an infectious disease like NE. In the particular case of chestnut and quebracho tannins, besides the inhibitory effects against *C*. *perfringens* and its toxins [10, 11], these phytochemicals display other multiple specific activities which can be related to improvement of growth efficiency and gut health [5, 13, 19, 36]. In addition, biological activities such as astringency, antioxidant [30, 37], increased epithelial cells division [37], intestinal microbiota modulation [13] and immunomodulation [38, 39], allow animals undergoing an infectious challenge which produces damage on the intestinal mucosa (i.e., Necrotic enteritis or coccidiosis), to recover faster, limiting the negative impacts on productive parameters.

The diverse array of biological effects of tannins have been shown to differ among animal species and to depend on time and dose exposure as well as on variations in chemical structures [40]. Based on their structural characteristics, tannins can be classified into two main groups: hydrolysable tannins (gallotannines, ellagitannines, gallic acid and ellagic derivatives) and condensed tannins (non-hydrolysable) called procyanidins, containing condensed carbon chain typical for flavonoids [40, 41]. Chestnut tannin (hydrolizable tannin), can be hydrolysed in the small intestine and be readily adsorbed by the mucosa, disappearing before reaching the distal gastrointestinal tract [42], modifying several cellular processes [37]. In contrast, quebracho tannin has lower bioavailability and *in vitro* antioxidant capacity than chestnut tannin [43], but these extracts contain mainly condensed tannins which are not easily hydrolysed, retaining most of their biological activities along the entire gastrointestinal tract although it could be metabolized in part by gut microbiota [19]. In the present work, the combination of both hydrolysable (chestnut) and condensed (quebracho) tannins in feed would influence homeostasis of intestinal epithelial cells, gut microbiota and physiological responses through a combination of direct and indirect effects, contributing to improve animal growth and health. Results from histological evaluation of small intestinal sections of AGP and tannin-supplemented animals showed differences in the intestinal epithelium between the two groups suggesting an effect of tannins on enterocytes as a result of both cytotoxic [30] and/or cytoprotective effects [37], which in addition may be different depending on bird age and intestinal segment. These apparent opposite actions imply that the potential benefits of tannins (and other phytochemicals) will depend on the effective concentrations of bioactive molecules that reach the epithelia as well as the presence or absence of metabolites produced by the resident microbiota influenced by these phytochemicals. Therefore, the correct doses of tannins supplemented to the feed seem to be highly important to produce beneficial effects in chickens, but it would be also depending on microbiota characteristics.

Many reports describe the role of gastrointestinal microbiota in host health and productive efficiency [44, 45], also most of these works relate modifications of resident microbial communities by feed additives such as AGPs or phytochemicals with the observed improvements in performance. Although results from previous studies show that antimicrobials reduce microbial diversity and tannins, as well as other phytochemicals, can produce opposite effects [13], in the present study no differences were observed between birds under AGP or tannin-based programs. These findings agree with previous studies from our group and others under experimental conditions describing cecal microbiota modulation by bacitracin and other AGPs [46–48]. Besides the lack of changes in global microbiota structure compared AGP vs tannins treated animals, the relative abundance of several taxa was different. Most bacterial groups increased in tannins supplemented flocks were previously associated with growth promoting effects such as *Lachnospiraceae* and *Ruminococcaceae* at the family level [44, 45], or *Lactobacillus* or *Bifidobacterium* at genus level [49], although correlations with productive efficiency were not found in the present study.

The experimental challenge with *C*. *perfringens* in an *in vivo* model of NE corroborated that the blend of tannins added to feed can significantly reduce intestinal lesions, suggesting a synergistic effect of chestnut and quebracho tannins, as the blend of these tannins was more efficient to control the occurrence of NE intestinal lesions than individual tannins. Also, the differences in the severity and distribution of intestinal gross lesions between animals fed with the commercial blend or the individual tannins suggest that each tannin provides differential activities to control NE. Following commercial blend, chestnut was the most effective additive to control NE, which could be explained in part by a direct and strong bactericidal effect [10] combined with differential activities along the different segments of the gastrointestinal tract as described before [19, 30, 37, 43]. While chestnut tannins can be hydrolysed in the small intestine and be readily absorbed by the mucosa, disappearing before reaching the distal gastrointestinal tract [42] with a probable reduction in their antimicrobial properties, derived metabolites may contribute to disease control through other cellular processes [37, 43], increasing epithelial resistance to pathogen actions and favouring recover. In contrast, quebracho extracts contain mainly condensed tannins which are not easily hydrolysed retaining their bacteriostatic activity against *C*. *perfringens* [10] along the entire gastrointestinal tract and probably in the faeces. The load of *C*. *perfringens* in faeces is highly reduced by quebracho tannins [10], preventing litter contamination and re-infection of chickens by faecal-oral route. The combination of hydrolysed and condensed tannins seems to assemble the individual benefits of each tannin group.

## Conclusion

Searching for reliable alternatives to AGPs has become a priority for the animal productive sector since several countries started limiting antibiotic use in animal production some years ago [4–7, 35, 50, 51]. Results from this study suggest that the use of tannin-based feed additives appears as an attractive alternative to promote gut health and productive efficiency. Additionally, necropsy observations during field trials and results from experimental *C*. *perfringens* challenge suggest that the blend of chestnut and quebracho tannins can help prevent and control NE with a reduction in the negative impact/consequences for poultry industry. Our data support the concept of replacing AGPs by phytochemical feed additives in commercial broiler flocks, preserving health and performance results, and suggests that this kind of additives can contribute to the implementation of AGP free programs. Moreover, since the farms enrolled in the present study followed management and vaccination procedures commonly used within the industry and furthermore, feed formulation did not differ from current industry standards, present results can be functional for most existing broiler chicken production systems.

## Supporting information

S1 File (DOCX)

S1 Fig Alpha diversity rarefaction curves. 16S rRNA gene V3V4 region amplicons were sequenced (Illumina MiSeq) and denoised with DADA2 software to obtain amplicon sequence variants (ASV). A and C show the Chao1 index (richness) per sample and per treatment, respectively. B and D show the Shannon’s index (entropy) per sample and per treatment, respectively. Boxes indicate SEM from 10 rarefaction iterations.
(TIF)

S2 Fig Cecal microbiota composition. Hierarchical taxonomy from phylum to species levels was assigned to ASVs using a naive Bayesian classifier trained on Green Genes 99% full-length 16S rRNA sequences in QIIME2 software. Each color represents a single taxon according to the legend to the right of the graphs. Each panel shows a different hierarchical classification level: (a) phylum; (b) family; (c) species. The taxa are ordered from higher (top) to lower (bottom) abundance.
(TIF)
