# Supplementary material for: Effects of a blend of chestnut and quebracho tannins on gut health and performance of broiler chickens
Source: PLoS One. 2022 Jan 21;17(1):e0254679. doi: 10.1371/journal.pone.0254679 (PMC8782372; doi:10.1371/journal.pone.0254679)
Supplement: S1 File — (DOCX) [file pone.0254679.s001.docx]

**Tannins and gut health in broilers:**

**Effects of a blend of chestnut and quebracho tannins on gut health and performance of broiler chickens**

Enzo A. Redondo ^1,2^, Leandro M. Redondo ^1,2^, Octavio A. Bruzzone ^2, 3^, Juan M. Diaz-Carrasco ^1,2^, Claudio Cabral ^4^, Victorino Garces ^5^, Maximo Liñeiro ^5^, Mariano E. Fernandez-Miyakawa ^1, 2 *^.

1 Instituto de Patobiología Veterinaria, Instituto Nacional de Tecnología Agropecuaria (INTA), Hurlingham, Buenos Aires, Argentina.

2 Consejo Nacional de Investigaciones Científicas y Técnicas (CONICET), Ciudad Autónoma de Buenos Aires, Argentina

3 EEA Bariloche, Instituto Nacional de Tecnología Agropecuaria, Bariloche, Río Negro, Argentina.

4 Silvateam S.A., Ciudad Autónoma de Buenos Aires, Argentina

5 Granja Tres Arroyos S.A., Capilla del Señor, Buenos Aires, Argentina

***Corresponding Author: fernandezmiyakawa.m@inta.gob.ar**

**Table S1:** Growth parameters derived from Gompertz equation (Experiment 2)

| **Parameters** | **AGPs** | **Tannins** | **p-values** |
| --- | --- | --- | --- |
| *Φ* | 0.800 | 0.661 | >0.05 |
| *εi* | 0.000 | 0.000 | >0.05 |
| *ɛs* | 0.094 | 0,098 | >0.05 |
| *R* | 0.094 | 0,089 | >0.05 |
| T (days) | 34.7 | 35.25 | >0.05 |
| *W_b_* (g) | 44.045 | 44.043 | >0.05 |
| *W_f_* (g) | 4437.678 | 4762.37 | >0.05 |
| T* (days) | 59.45 | 58.8 | >0.05 |

*Φ*: Aurorregressive coefficient; *εi:* Error intercept; *ɛs*: Error Slope; R: growth rate; T: age at which growth rate is maximized; W_b_: initial weight; W_f_: Weight at maturity; T*: age to reach Wf.

**Table S2:** Structure of the cecal microbiota, AGPs vs. tannins (Experiment 2)

|  | **AGPs** | **Tannins** | ***p* values** | **r values** | | |
| --- | --- | --- | --- | --- | --- | --- |
|  |  |  |  | **BW** | **FCR** | **EPEF** |
| Number of ASVs  (Richness) | 264.7 ± 52.36 | 281.6 ± 57.96 | 0.427 | 0.350 | 0.57 * | - 0.8* |
| Shannon’s index | 7.41 ± 0.2225 | 7.46 ± 0.2175 | 0.470 | 0.368 | 0.56 * | -0.77* |
| Simpson’s Eveness  Index | 4.671 ± 1.115 | 3.409 ± 1.009 | 0.264 | -0.34 | - 0.53 * | 0.81* |
| Faith’s index | 18.25 ± 2.089 | 18.43 ± 1.462 | 0.398 | 0.437 | 0.6 * | 0.78* |
| Firmicutes/Bacteroidetes ratio | 6.248 ± 1.582 | 7.171 ± 2.586 | 0.485 | 0.254 | - 0.43 | 0.36 |

Values are expressed as the means ± standard errors.

BW: body weight; FCR: feed conversion ratio; EPEF: European Poultry Efficiency Factor.

* p<0.05

**Table S3:** Frequency of severe intestinal gross lesions^1^ (Experiment 3)

| Treatment | Jejunum (%) | Ileum (%) |
| --- | --- | --- |
| Positive control | 25.81 | 25.81 |
| Chestnut | 16.67 | 8.33 |
| Quebracho | 6.67 | 10.00 |
| Tannins mix | 12.5 | 6.25* |

*: p<0.05

^1^ Intestinal gross lesion with score >3 were classified as severe. Score 0: no apparent gross lesions; 1: removable fibrin deposit; 2: isolated focal necrosis or ulceration (1 to 5 foci); 3: multiple focal necrosis or ulceration (6 or more foci); 4: extensive areas of necrosis; 5: diffuse necrosis, presence of attached pseudomembrane).

**Figure legends:**

**Fig S1: Alpha diversity rarefaction curves.** 16S rRNA gene V3V4 region amplicons were sequenced (Illumina MiSeq) and denoised with DADA2 software to obtain amplicon sequence variants (ASV). A and C show the Chao1 index (richness) per sample and per treatment, respectively. B and D show the Shannon’s index (entropy) per sample and per treatment, respectively. Boxes indicate SEM from 10 rarefaction iterations.

**Fig S2: Cecal microbiota composition.** Hierarchical taxonomy from phylum to species levels was assigned to ASVs using a naive Bayesian classifier trained on Green Genes 99% full-length 16S rRNA sequences in QIIME2 software. Each color represents a single taxon according to the legend to the right of the graphs. Each panel shows a different hierarchical classification level: (a) phylum; (b) family; (c) species. The taxa are ordered from higher (top) to lower (bottom) abundance.
