# Supplementary figures and images for: Effects of a blend of chestnut and quebracho tannins on gut health and performance of broiler chickens
Source: PLoS One. 2022 Jan 21;17(1):e0254679. doi: 10.1371/journal.pone.0254679 (PMC8782372; doi:10.1371/journal.pone.0254679)

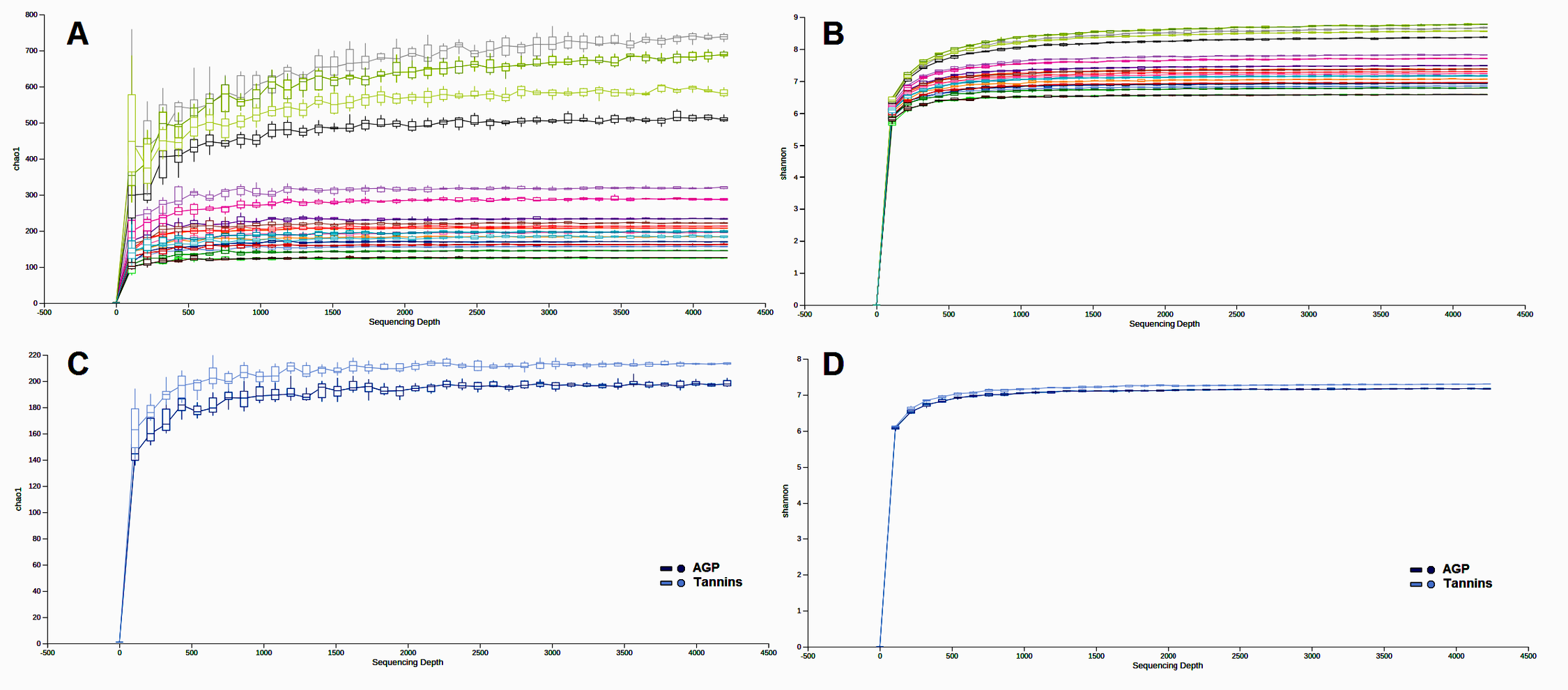

Supplement: S1 Fig — 16S rRNA gene V3V4 region amplicons were sequenced (Illumina MiSeq) and denoised with DADA2 software to obtain amplicon sequence variants (ASV). A and C show the Chao1 index (richness) per sample and per treatment, respectively. B and D show the Shannon’s index (entropy) per sample and per treatment, respectively. Boxes indicate SEM from 10 rarefaction iterations. (TIF) [file pone.0254679.s002.tif]

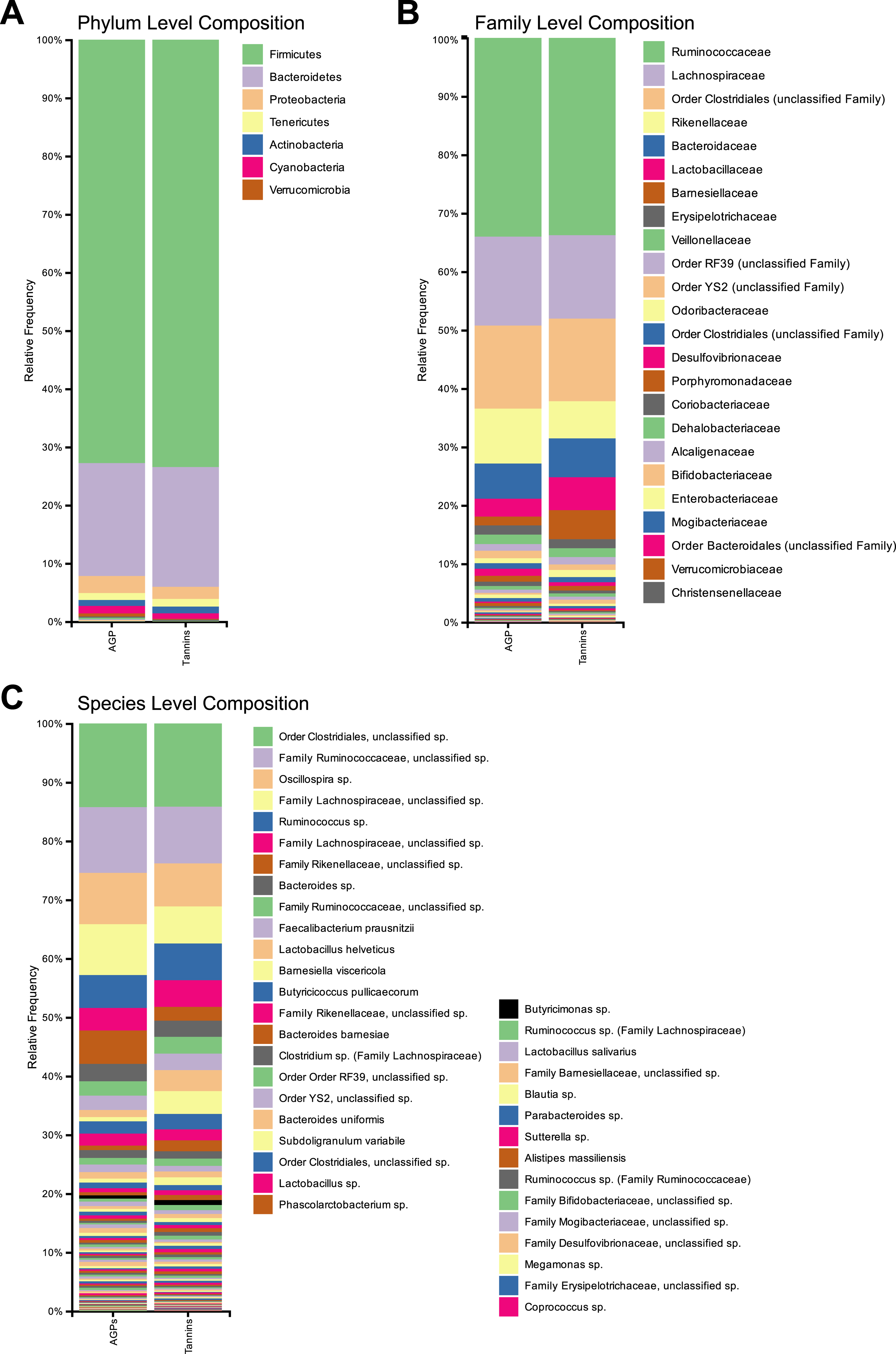

Supplement: S2 Fig — Hierarchical taxonomy from phylum to species levels was assigned to ASVs using a naive Bayesian classifier trained on Green Genes 99% full-length 16S rRNA sequences in QIIME2 software. Each color represents a single taxon according to the legend to the right of the graphs. Each panel shows a different hierarchical classification level: (a) phylum; (b) family; (c) species. The taxa are ordered from higher (top) to lower (bottom) abundance. (TIF) [file pone.0254679.s003.tif]
